# Optimal duration of anticoagulant thromboprophylaxis in total hip arthroplasty: new evidence in 55,540 patients with osteoarthritis from the Nordic Arthroplasty Register Association (NARA) group

**DOI:** 10.1080/17453674.2019.1611215

**Published:** 2019-05-07

**Authors:** Alma B Pedersen, Ina Trolle Andersen, Soren Overgaard, Anne Marie Fenstad, Stein Atle Lie, Jan-Erik Gjertsen, Ove Furnes

**Affiliations:** a Department of Clinical Epidemiology, Aarhus University Hospital, Denmark;; b Department of Orthopaedic Surgery and Traumatology, Odense University Hospital, Department of Clinical Research, University of Southern Denmark, Odense, Denmark;; c The Norwegian Arthroplasty Register, Department of Orthopedic Surgery, Haukeland University Hospital, Bergen, Norway;; d Department of Clinical Medicine, University of Bergen, Norway;; e Department of Clinical Dentistry, University of Bergen, Bergen, Norway

## Abstract

Background and purpose — The recommended optimal duration of the thromboprophylaxis treatment in total hip arthroplasty (THA) patients has been a matter of debate for years. We examined the association between short (1–5 days), standard (6–14 days), and extended (≥ 15 days) duration of thromboprophylaxis, with regards to the risk of venous thromboembolism (VTE), major bleeding, and death in unselected THA patients.

Patients and methods — We performed a cohort study using prospectively collected data from the population-based hip arthroplasty registries, prescription databases, and patient administrative registries in Denmark and Norway. We included 55,540 primary THA patients with osteoarthritis

Results — The 90-day cumulative incidence of VTE was 1.0% for patients with standard treatment (reference), 1.1% for those with short-term treatment (adjusted hazard ratio [aHR] of 1.1, 95% confidence interval (CI) 0.8–1.5) and 1.0% for those with extended treatment (aHR of 0.9, CI 0.8–1.2). The aHRs for major bleeding were 1.1 (CI 0.8–1.6) for short and 0.8 (CI 0.6–1.1) for extended vs. standard treatment. In addition, patients with short and extended treatment had aHRs for death of 1.2 (CI 0.8–1.8) and 0.8 (CI 0.5–1.1) vs. standard treatment, respectively. Patients who started short treatment postoperatively had an aHR for death of 1.8 (CI 1.1–3.1) and absolute risk difference of 0.2%, whereas patients who started short treatment preoperatively had an aHR for death of 0.5 (CI 0.2–1.2) and absolute risk difference of 0.3% compared with patients who had standard treatment with post- and preoperative start, respectively.

Interpretation — In routine clinical practice, we observed no overall clinically relevant difference in the risks of VTE and major bleeding within 90 days of THA with respect to thromboprophylaxis duration. However, our data indicate that short-term thromboprophylaxis started postoperatively is associated with increased 90-day mortality. The significance of these data should be explored further.

The incidence of total hip arthroplasty (THA) procedures increases annually worldwide (Nemes et al. 2014). Risk of symptomatic venous thromboembolism (VTE) within 90 days of THA are reported to range from 1% to 4% (Pedersen et al. 2012, Huo 2012, Wolf et al. 2012) in the presence of thromboprophylaxis, and is furthermore elevated up to 1 year postoperatively (Pedersen et al. 2012). Given the high risk of VTE in the absence of thromboprophylaxis and high mortality following symptomatic VTE (Pedersen et al. 2017), anticoagulant thromboprophylaxis for THA patients is preferred treatment in most countries. However, the recommended optimal duration of the treatment has been a matter of debate for years.

The American College of Chest Physicians (ACCP) guidelines from 2012 recommend a minimum of 10 to 14 days of thromboprophylaxis and suggest extending the treatment to 35 days in the outpatient period (Falck-Ytter et al. 2012). The American Academy of Orthopaedic Surgeons (AAOS) guidelines from 2011 recommend individual assessment of the optimal duration of thromboprophylaxis (AAOS 2013). Since a number of concerns have been identified with these guidelines (Budhiparama et al. 2014), and due to considerable change in the THA course with introduction of fast-track programs in orthopedic departments, several national guidelines have been published since. Danish national guidelines recommend anticoagulant thromboprophylaxis for 6–10 days in THA patients, and less than 5 days if fast-track THA surgery was performed (Danish Council for the Use of Expensive Hospital Medicine [RADS] 2016). In Norway, thromboprophylaxis is recommended for 10 postoperative days (Granan 2015). The latest paper from the Cochrane database of systematic reviews concluded that there is moderate quality evidence for extended duration of thromboprophylaxis to prevent VTE in THA patients (Forster and Stewart 2016). Neither of the guidelines suggests risk stratification in order to provide specific duration of thromboprophylaxis for specific THA patients. A Danish cohort study observed no overall difference in the risk of VTE or bleeding with respect to thromboprophylaxis duration in THA patients from routine clinical practice (Pedersen et al. 2015), but this study lacked statistical power to analyze data on the subgroup level.

We examined the association between duration of anticoagulant thromboprophylaxis for the prevention of VTE in patients undergoing elective THA in Denmark and Norway. As a safety outcome, we consider bleeding and death. We also aimed to identify THA patients who could benefit from extended prophylaxis without increase in bleeding events.

## Patients and methods

### Study design and setting

We conducted this population-based cohort study using prospectively collected data available from the Nordic Arthroplasty Register Association (NARA) database, established in 2009. All Swedish, Norwegian, Danish, and Finnish citizens are assigned a unique civil registration number, permitting unambiguous linkage between hip registries and other medical databases in each country. This also enables tracking of deceased and emigrated patients (Schmidt et al. 2014). The healthcare system in Scandinavian countries provides tax-supported healthcare for all citizens; free medical care is guaranteed for emergency and general hospital admissions, as well as for outpatient clinic visits. The study is reported according to the RECORD guidelines.

### Study population

We used the NARA database to identify all patients operated in Denmark and Norway. Data from Sweden and Finland were not included, since individual-level data on duration of anticoagulant thromboprophylaxis were not available from these countries. We included all primary THAs between January 1, 2010 and December 31, 2014 from Denmark (n = 34,625) and between January 1, 2008 and December 31, 2013 from Norway (n = 34,801), and accessed their preoperative and surgery-related records. Primary THA was defined as insertion of a unilateral total hip prosthesis due to primary osteoarthritis.

We excluded 269 and 261 patients from Denmark and Norway, respectively, since these were registered in NARA as not receiving any anticoagulant thromboprophylaxis, leaving 34,356 Norwegian and 34,540 Danish THAs in the study population. We restricted the study population to first-time THA due to primary OA, leaving 26,250 THA patients in Denmark and 31,096 patients in Norway. We excluded 1,806 patients from Norway due to registration of several thromboprophylaxis drugs as the first choice. Finally, 2,310 procedures in Denmark and 2,748 in Norway lacked information on duration of thromboprophylaxis, although they were registered as receiving thromboprophylaxis, which resulted in 23,940 and 26,542 procedures for the complete-case analyses.

### Duration of anticoagulant thromboprophylaxis

We categorized the duration of anticoagulant thromboprophylaxis prescribed by surgeon in relation to THA surgery as short-term (1–5 days), standard (6–14 days), or extended (≥ 15 days) based on available international guidelines for thromboprophylaxis and clinical practice in Denmark and Norway. Allocation of duration of treatment is dependent on the local guidelines at the individual departments. Hence, most standard patients operated at one department will receive the same length of treatment irrespective of their risk. However, high-risk patients such as those with previous VTE or cancer have a higher likelihood of receiveing extended prophylaxis. We included the following anticoagulant agents approved for use in the Denmark and Norway for prevention of VTE in THA patients: parenteral low-molecular-weight heparin (including enoxaparin, dalteparin, and tinzaparin) and fondaparinux, dabigatran, and rivaroxaban, initiated both pre- and postoperatively. Mechanical thromboprophylaxis, when used, is combined with anticoagulant thromboprophylaxis.

### Outcome

The outcomes of interest were VTE, major bleeding, and death. These data were obtained from the Danish National Patient Registry (DNRP) (Schmidt et al. 2015) and the Norwegian Patient Register (NPR) (Bakken et al. 2004). The effectiveness outcome in our analyses was VTE, including deep venous thrombosis (DVT) and pulmonary embolism (PE), whereas safety outcome was death and major bleeding events, including intracranial bleeding, gastrointestinal bleeding, and urinary/lung bleeding. The proportion of hospitalized patients correctly registered in the DNRP and NPR with cardiovascular events and major bleeding has been reported as 75% to 95% (Adelborg et al. 2016, Sundboll et al. 2016). Both primary and secondary diagnoses, which were coded by the physician at the discharge from the hospital or during the outpatient visit, were included in our study.

Outcome data from general practitioners were not available. Since any suspicion of VTE and major bleeding would lead to hospitalization or outpatient clinic visit of the majority of patients to confirm the diagnosis and initiate treatment, we may lacked only few patients from general practitioners.

Information on death, available from the Danish Civil Registration System (Schmidt et al. 2014) and Statistics Norway, was linked to each patient in the national registers before transferring the data to NARA.

### Covariates

Information on comorbidity was collected from the DNRP and NPR before transferring to the NARA database. For all included THA patients, we identified all primary and secondary discharge diagnoses for all hospitalizations and hospital specialist outpatient visits that occurred 1 year prior to the time of surgery. We computed the Charlson Comorbidity Index (CCI) score for each patient at the time of surgery. We defined 3 comorbidity levels: a score of 0 (low), given to patients with no previous record of diseases included in the CCI; a score of 1–2 (medium); and a score of 3 or more (high). Further variables were included as confounders: age and sex at the time of THA, year of primary THA, fixation type (cemented, uncemented, and hybrid/inverse hybrid fixation), and start of thromboprophylaxis (pre- or postoperative). In addition, we obtained information on the use of acetylsalicylic acid (both low-dose and high-dose), vitamin K antagonists, and platelet inhibitors (clopidogrel, prasugrel, and ticagrelor) from the Danish National Database of Reimbursed Prescriptions (Johannesdottir et al. 2012) and Norwegian Prescription Database (Furu 2008) during the 2 years preceding the primary THR.

### Statistics

We used the cumulative incidence function to compute the 90-day risk of VTE, major bleeding, and death, considering death as competing risk. We used Cox regression to compute crude and hazard ratios (HRs) adjusted for potential confounding factors with 95% confidence intervals (CIs). By application of log–log plots and Cox proportional hazards, the statistical model was found suitable. Follow-up started on the day of primary THA and ended either on the day of discharge from the primary hospitalization (if outcome occurred in the hospital), on the day of readmission for VTE/bleeding, date of death, emigration, or 90 days after surgery, whichever came first. All analyses were repeated stratifying on age, sex, CCI, thromboprophylaxis start, and country of origin in order to study the potential difference in effect of duration of thromboprophylaxis on outcomes in subgroups of patients.

Patients were included in each exposure category according to initial treatment assignment. Therefore, in the main analyses we started follow up at the time of THA surgery to examine the effect of initial assigned treatment on VTE/bleeding/death risk (according to intent to treat approach).

### Sensitivity analyses

We tested the robustness of the results in a series of sensitivity analyses. 1st, the risk of all-cause mortality was calculated. 2nd, the risk of VTE and all-cause mortality defined as any DVT, non-fatal PE or all-cause mortality was calculated. 3rd, we analyzed data only for patients that were alive at day 7 or day 14 post-surgery without any VTE event during the first 7 or 14 postoperative days. These data will mimic randomization start in some clinical trials, which compares extended prophylaxis with placebo given after the 7th day (Eikelboom et al, 2001). In addition, these analyses should contribute to understanding of immortal time bias. 4th, we changed exposure definition to short-term (1–7 days), standard (8–14 days), or extended (≥ 15 days), since some hospitals provide prophylaxis using weeks as guideline. 5th, we ignored revision as a competing risk. 6th, we performed cluster analyses at hospital level in order to consider unmeasured hospital specific confounders. 7th, we analyzed data only for patients without use of acetylsalicylic acid before surgery. Analyses were performed using SAS V. 9.4 (SAS Institute Inc., Cary, NC, USA).

All diagnosis codes used to identify VTE and major bleeding after THA are presented in Appendix 1 (see Supplementary data).

### Ethics, funding, and potential conflicts of interest

The study was approved by the Danish Data Protection Agency (Region of Central Denmark Jr. nr. 1-16-02-743-14) and Regional Ethical Committee of western Norway (Ref. 2015/880/REK Vest). No funding was received for this study and the authors report no conflicts of interest.

## Results

Among the 55,540 THA patients operated in Denmark during 2010–2014 and Norway during 2008–2013, 15% received short, 31% received standard, and 45% received extended thromboprophylaxis treatment. Data regarding duration of treatment were missing in 9% (Table 1).

**Table 1.** Patient characteristics, medication use, and surgery-related factors of 55,540 patients undergoing THA in Denmark in 2010–2014 and Norway in 2008–2013

Duration of thromboprophylaxis					
Factor	Missing	Short	Standard	Extended	Total
	n (%)	n (%)	n (%)n (%)	n (%)	n (%)
Total	5,058 (100)	8,333 (100)	17,009 (100)	25,140 (100)	55,540 (100)
Age group					
10–59	757 (15)	1,487 (18)	2,790 (16)	3,811 (15.2)	8,845 (16)
60–69	1,670 (33)	2,819 (34)	5,646 (33.2)	8,665 (35)	18,800 (34)
70–79	1,877 (37)	2,890 (35)	6,129 (36)	8,971 (38)	19,867 (36)
80+	754 (15)	1,137 (14)	2,444 (14)	3,693 (15)	8,028 (15)
Sex					
Female	2,967 (59)	4,586 (55)	10,326 (61)	15,975 (64)	33,854 (61)
Male	2,091 (41)	3,747 (45)	6,683 (39)	9,165 (36)	21,686 (39)
Charlson Comorbidity Index					
Missing data	27 (0.5)	2 (0)	26 (0.2)	215 (1)	270 (1)
Low	4,158 (82)	7,352 (88)	14,175 (83)	20,371 (81)	46,056 (83)
Medium	770 (15)	864 (10)	2,486 (15)	3,975 (16)	8,095 (15)
High	103 (2)	115 (1)	322 (2)	579 (2)	1119 (2)
Acetylsalicylic acid					
No	3,815 (75)	6,594 (79)	13,108 (77)	19,202 (76)	42,719 (77)
Yes	1,243 (25)	1,739 (21)	3,901 (23)	5,938 (24)	12,821 (23)
Clopidogrel/prasugrel/ticagrelor					
No	4,949 (98)	8,107 (97)	16,635 (98)	24,727 (98)	54,418 (98)
Yes	109 (2)	226 (3)	374 (2)	413 (2)	1,122 (2)
Marevan/marcoumar/dabigratran/apixaban/rivaroxaban					
No	4,550 (90)	7,699 (92)	15,764 (93)	23,970 (95)	51,983 (94)
Yes	508 (10)	634 (8)	1,245 (7)	1170 (5)	3557 (6)
Type of fixation					
Missing data	72 (1)	16 (0)	178 (1)	343 (1)	609 (1)
Cementeret	1,530 (30)	481 (6)	5,164 (30)	8,971 (36)	16,146 (29)
Uncementeret	1,989 (39)	5,934 (71)	8,625 (51)	9,123 (36)	25,671 (46)
Hybrid	491 (10)	1,691 (20)	855 (5)	774 (3)	3,811 (7)
Reverse hybrid	976 (19)	211 (3)	2,187 (13)	5,929 (24)	9,303 (17)
Start of thromboprophylaxis					
Missing data	1,056 (21)	51 (1)	555 (3)	1,866 (7)	3,528 (6)
Preoperative	1,293 (26)	2,677 (32)	4,892 (29)	6,334 (25)	15,196 (27)
Postoperative	2,709 (54)	5,605 (67)	11,562 (68)	16,940 (67)	36,816 (66)

Compared with patients who received standard duration of treatment, patients with short duration of treatment were slightly younger, included fewer females, included fewer patients with CCI score medium and high, included fewer patients who received acetylsalicylic acid but more patients who received vitamin K antagonists and platelet inhibitors, and were more likely to undergo uncemented or hybrid THA in Denmark. On the other hand, patients with extended duration of treatment were also slightly younger, included more females, included more patients with CCI score medium and high, included fewer patients who received vitamin K antagonists and platelet inhibitors, and were less likely to undergo uncemented or hybrid THA.

In the group with short duration of treatment, 8% were prescribed prophylaxis for 1 day, 20% were prescribed treatment for 2 days, 34% of patients were prescribed prophylaxis for 3 days, 17% were prescribed treatment for 4 days, and 17% were prescribed treatment for 5 days. In the group with standard duration of thromboprophylaxis, 47% of patients were prescribed treatment for 7 days whereas 22% and 24% were prescribed treatment for 10 and 14 days, respectively. In the group with extended treatment duration, 12%, 20%, and 46% of patients received thromboprophylaxis for 28 days, 30 days, and 35 days, respectively. In the group with short-term treatment duration, 44% received low-molecular-weight heparin, compared with 86% and 71% in the group with standard treatment and extended duration of treatment.

Within 90 days of surgery, cumulative incidences of VTE were 1.1% for patients prescribed short-term thromboprophylaxis, and 1.0% for both standard and extended treatment (Figure). This correspond to the adjusted HRs for VTE of 1.1 (CI 0.8–1.5) and 0.9% (CI 0.8–1.2) for patients prescribed short-term and extended thromboprophylaxis compared with standard thromboprophylaxis treatment (Table 2).

**Table 2.** Effect of thromboprophylaxis treatment duration on the risk of death, thromboembolism (VTE), major bleeding, VTE, and death, and risk of revision in total hip arthroplasty patients within 90 days of surgery, with standard treatment as the reference

Outcome	Duration of thrombo- prophylaxis	Outcome (n)	Number at risk	Crude HR (95% CI)	Adjusted HR (95% CI)	90 days cumulative incidence (95% CI)
Death	Short	39	8,333	1.3 (0.9–1.9)	1.2 (0.8–1.8)	0.5% (0.3–0.6)
Death	Standard	62	17,009	1.0	1.0	0.4% (0.3–0.5)
Death	Extended	72	25,140	0.8 (0.6–1.1)	0.8 (0.5–1.1)	0.3% (0.2–0.4)
VTE	Short	89	8,333	1.0 (0.8–1.3)	1.1 (0.8–1.5)	1.1% (0.9–1.3)
VTE	Standard	176	17,009	1.0	1.0	1.0% (0.9–1.2)
VTE	Extended	245	25,140	0.9 (0.8–1.1)	0.9 (0.8–1.2)	1.0% (0.9–1.1)
Bleeding	Short	56	8,333	1.0 (0.7–1.4)	1.1 (0.8–1.6)	0.7% (0.5–0.9)
Bleeding	Standard	116	17,009	1.0	1.0	0.7% (0.6–0.8)
Bleeding	Extended	128	25,140	0.7 (0.6–1.0)	0.8 (0.6–1.1)	0.5% (0.4–0.6)
VTE/death	Short	125	8,333	1.1 (0.9–1.4)	1.1 (0.9–1.4)	1.5% (1.3–1.8)
VTE/death	Standard	233	17,009	1.0	1.0	1.4% (1.2–1.6)
VTE/death	Extended	315	25,140	0.9 (0.8–1.1)	0.9 (0.8–1.1)	1.3% (1.1–1.4)

HR = hazard ratio; CI = confidence interval.

HR adjusted for age, sex, Charlson Comorbidity Index score, type of fixation, start of thromboprophylaxis, as well as acetylsalicylic acid, clopidogrel/prasugrel/ticagrelor, marevan/marcumar/dabigatran/apixaban/rivaroxaban used before surgery.

Cumulative incidences of VTE/death as one outcome within 90 days were 1.5% for patients prescribed short-term thromboprophylaxis, 1.4% for standard treatment duration, and 1.3% for extended treatment duration. The adjusted HRs for VTE/death were 1.1 (CI 0.9–1.4) for patients prescribed short-term and 0.9 (CI 0.8–1.1) for extended treatment, compared with standard thromboprophylaxis treatment.

Within 90 days of surgery, cumulative incidences of major bleeding were 0.7% for patients treated with both short and standard treatment, and 0.5% for extended thromboprophylaxis treatment (Figure). The adjusted HRs for major bleeding were 1.1 (CI 0.8–1.6) for short-term treatment and 0.8 (CI 0.6–1.1) for extended duration of treatment compared with standard treatment.

The risk of death within 90 days of surgery was 0.5% among patients prescribed short-term treatment, 0.4% for standard treatment, and 0.3% for extended treatment. The adjusted HRs for death were 1.2 (CI 0.8–1.8) for short-term duration of treatment, and 0.8 (CI 0.5-1.1) for extended duration of treatment, compared with standard duration of treatment.

Analyses stratified on country of origin showed similar results to those of the overall population for all outcomes (Appendix 2, available from the authors). Stratification on sex showed that females receiving short treatment duration were associated with increased risk of VTE and VTE/death (adjusted HR were 1.6, CI 1.1–2.2 and 1.6, CI 1.2–2.2) compared with standard treatment. However, absolute risk differences were 0.4% and 0.5%, respectively. No increased risk for VTE and VTE/death was observed for male patients with short treatment duration, or for female/male patients receiving extended treatment compared with standard treatment. Stratification on different age and CCI score groups produced the estimates similar to overall risk estimates for any outcome.

Stratification on start of thromboprophylaxis showed that short treatment started postoperatively was associated with increased risk of dying (adjusted HR was 1.8, CI 1.1–3.1 and the absolute risk difference was 0.2%), whereas short treatment started preoperatively was associated with reduced risk of dying (adjusted HR was 0.5, CI 0.2–1.2 and the absolute risk difference was 0.3%) compared with patients with standard treatment with post- and preoperative start, respectively. No difference in risk of dying was observed for patients starting extended treatment either post- or preoperatively. The adjusted HR for dying for short treatment started postoperatively vs. standard treatment started postoperatively was 2.2 (CI 1.1–4.2) among patients without use of acetylsalicylic acid before surgery.

**Figure:**
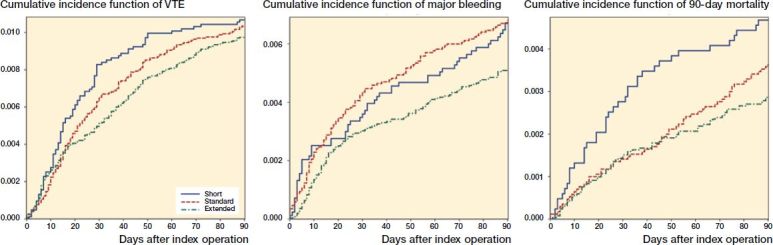
Cumulative incidence function of venous thromboembolism (VTE), major bleeding, and mortality within 90 days of total hip arthroplasty.

We found similar risk estimates derived from the cluster analyses and those derived from analyses without clustering for hospitals (data not shown). In addition, analyses with slight change of exposure categories definition, analyses not including revision as competing risk into appropriate models, or analyses among THA patients who were alive at day 7/14 without any VTE event during the period from the day of primary THA to 7/14 days after, did not affect overall study results (data not shown).

## Discussion

In this large population-based study including 55,540 THA patients from routine clinical practice, we found no overall difference in risk of VTE, VTE/death, or major bleeding with respect to the duration of pharmacological thromboprophylaxis. However, differences between duration of treatments were observed when stratifying on sex and start of thromboprophylaxis treatment, suggesting that female patients with short treatment have increased risk of VTE and patients with short treatment initiated postoperatively have increased risk of dying. This could be used to guide preventive treatment for subgroups of patients; however, low absolute risk difference estimates should be taken into consideration.

### Strengths and weaknesses

Completeness and validity of data in the Danish Hip Arthroplasty Registry and Norwegian Arthroplasty Register are documented to be more than 95% (Pedersen et al. 2004, Arthursson et al. 2005). The positive predictive value of VTE and major bleeding is 75–95% with an accuracy of 94–100% of the comorbid diagnoses included in the CCI (Bakken et al. 2004, Thygesen et al. 2011, Sundboll et al. 2016). Due to prospective registration of data, misclassification of VTE/bleeding is not likely to be related to registration of exposure status (thus, duration of treatment). Therefore, our HRs should be unbiased. However, our absolute cumulative incidences of VTE/bleeding are underestimated. Information on death and migration was available from the Civil Registration System, allowing for nationwide cohort studies with virtually complete follow-up (Schmidt et al. 2014). Further, selection bias in our study is low since we included all THA patients treated in many departments irrespective of specific patient characteristics or willingness to participate in the study.

We lack data on duration of treatment for 9% of patients. However, the median age, sex, and comorbidity level for the excluded patients were similar to those of patients included in the study population. In light of prospective collection of data on duration of treatment and subsequent outcomes of interest, it is unlikely that missing data on duration of treatment occurred systematically. Hypothetically, if patients’ compliance with the treatment after discharge had changed, which is most worrisome for patients included in the extended treatment group, this could have affected our results in any direction. Although we were able to adjust for a number of potential confounders, including pre-admission anticoagulant medication, unmeasured and residual confounding, as well as confounding by indication, cannot be accounted for entirely in an observational study, in particular when studying the effect of treatment. We have, however, performed a number of sensitivity analyses in order to elucidate any confounding bias.

We have used the cumulative incidence method to estimate crude failure (crude VTE or crude bleeding risk), which is the likely number of VTE or bleeding incidents we see in clinical practice and consists of both the failure of the VTE/bleeding and the mortality process (Sayers et al. 2018). In Table 2 we present both number of failures (VTE/bleeding) and number of patients at risk, which allows for estimation of net failure (the Kaplan–Meier estimates with immortal patients). It is worth noting that the difference between cumulative incidence and Kaplan–Meier estimates is very small, most likely due to short-term follow-up and low mortality in the exposure groups.

### Comparison with other studies

2 recently published systematic reviews based on clinical trials (Forster and Stewart 2016, Trevisol et al. 2018) both concluded that extended duration thromboprophylaxis should be considered to prevent VTE, when undergoing THA. Forster et al. furthermore specified that the evidence for extended duration was moderate, and that the benefit should be weighed against the increased risk of minor bleeding. Likewise, 2 systematic reviews based on older clinical trials (Hull et al. 2001, Sobieraj et al. 2012) also concluded that extended thromboprophylaxis is more efficacious than shorter thromboprophylaxis for preventing VTE or VTE-related deaths while increasing the risk of minor bleeding. These results do not correlate with our findings.

However, in a systemic review, O’Donnell et al. (2003) concluded that the absolute reduction in symptomatic VTE attributed to extended prophylaxis in some clinical trials seems to have been overestimated. The authors suggested that the assessment of symptomatic VTE in clinical trials did not occur independently of the routine venography results, which could have led to an overestimation of the symptomatic VTE risk and the absolute risk reduction attributed to extended prophylaxis. A prospective, observational, non-interventional, phase IV study of 3,914 consecutive patients who underwent total hip or knee arthroplasty during 2010 to 2012 reported that treatment with rivaroxaban for 15 days was protective against symptomatic VTE without excess risk of major bleeding (Gomez et al. 2017). The absolute risk of VTE and bleeding in that study was 0.5% and 0.3%, respectively. In the same study, the authors calculated that treating all 1,444 primary THA patients for 35 days with rivaroxaban could theoretically prevented 1–3 symptomatic proximal DVTs. Based on 16,865 THA patients operated from 2010 through 2012 in Denmark, we have previously reported that there is no difference in the risk of VTE and bleeding with respect to duration of thromboprophylaxis (Pedersen et al. 2015). A study including only patients from fast-track departments where thromboprophylaxis is provided for 1–2 days reported an absolute risk of VTE within 90 days of 0.5% (Petersen et al. 2018). These absolute VTE risk estimates are similar to estimates observed in the current study and estimates from clinical trials.

Extended prophylaxis is further challenged by aspirin as potential prophylaxis treatment. Azboy et al. (2017) performed a review on aspirin studies, concluding that there is convincing evidence that aspirin is an effective, inexpensive and safe VTE prevention treatment in THA patients without increased risk of bleeding. Safety of aspirin was reported when administered as part of a hybrid strategy that follows a short course of anticoagulation in select low-risk populations, but also when administered alone in appropriately screened patients (Anderson et al. 2018). Since the rates of VTE and bleeding are very low, the potential benefits of extended prophylaxis may not be worth discussing. The orthopedic surgeons are currently discussing not how long, but how short a duration of thromboprophylaxis should be given, targeting extended thromboprophylaxis to high-risk patients.

Our stratified analyses suggest that the start of thromboprophylaxis might play a role since patients who started short treatment postoperatively had an increased risk of dying. This is in line with the latest findings from Norway, showing that THA patients who started thromboprophylaxis preoperatively vs. those who started postoperatively have adjusted odds ratio for dying of 0.7 (CI 0.5–1.1), whereas the risk of other postoperative complications was similar in the two groups (Borgen et al. 2017).

## Conclusions

In this large cohort study including 55,540 THA patients from a routine clinical practice in Denmark and Norway, we found no overall clinically relevant difference in risk of VTE and bleeding with respect to the duration of pharmacological thromboprophylaxis. However, higher relative risk of dying for short treatment initiated postoperatively was observed. The significance of these data should be explored further, and short-term thromboprophylaxis started preoperatively should be considered.

## Supplementary data

Appendix 2. Stratified analyses: cohort of first primary THA operation in Denmark in 2010–2014 and Norway in 2008–2013 stratified on age groups, CCI, country, sex, and start preoperatively versus postoperatively is available on request from the authors.

Appendix 1 is available as supplementary data in the online version of this article, http://dx.doi.org/10.1080/17453674. 2019.1611215

ABP, SO, and OF conceived the idea for this study. ITA performed the analyses. All co-authors participated in drafting and revising the paper, data design, data interpretations and conclusions, and final approval of the paper. ABP and OF take responsibility for the integrity of the work.

*Acta* thanks Banne Nemeth for help with peer review of this study.

AAOS Preventing venous thromboembolic disease in patients undergoing elective hip and knee arthroplasty. Evidence-based guideline and evidence report. http://www.aaos.org/research/guidelines/VTE/VTE_full_guideline.pdf; 2013 (last accessed February 14, 2019).

## Supplementary Material

Supplemental Material
