# Supplementary material for: Optimal duration of anticoagulant thromboprophylaxis in total hip arthroplasty: new evidence in 55,540 patients with osteoarthritis from the Nordic Arthroplasty Register Association (NARA) group
Source: Acta Orthop. 2019 May 7;90(4):298–305. doi: 10.1080/17453674.2019.1611215 (PMC6718178; doi:10.1080/17453674.2019.1611215)
Supplement: Supplemental Material [file IORT_A_1611215_SM3019.pdf]

Supplementary data

Appendix 1. Codes from the International Classification of Diseases, 10th edition (ICD-10), used to identify study outcomes in the Danish National Registry of Patients

Study outcome	ICD-10 codes
Venous thromboembolism, deep venous thrombosis, and pulmonary embolism	I26, I80.1–I80.9 and I82.1–I82.9
Intracranial bleeding	I60- I62, I690–I692 and S064–S066
Gastrointestinal bleeding	K25.0, K25.2, K25.4, K25.6, K26.0, K26.2, K26.4, K26.6, K27.0, K27.2, K27.4, K27.6, K28.0, K28.2, K28.4, K28.6, K29.0, and K92.0–K92.2
Urinary/lung bleeding	J942, N02, R04, R31
